# Use of PTC124 for nonsense suppression therapy targeting *BMP4* nonsense variants *in vitro* and the *bmp4*^st72^ allele in zebrafish

**DOI:** 10.1371/journal.pone.0212121

**Published:** 2019-04-24

**Authors:** Max Krall, Stephanie Htun, Anne Slavotinek

**Affiliations:** Division of Genetics, Department of Pediatrics, University of California San Francisco, San Francisco, CA, United States of America; Lee Kong Chian School of Medicine, SINGAPORE

## Abstract

Nonsense suppression therapy (NST) utilizes compounds such as PTC124 (Ataluren) to induce translational read-through of stop variants by promoting the insertion of near cognate, aminoacyl tRNAs that yield functional proteins. We used NST with PTC124 to determine if we could successfully rescue nonsense variants in human Bone Morphogenetic Protein 4 (*BMP4*) *in vitro* and in a zebrafish *bmp4* allele with a stop variant *in vivo*. We transfected 293T/17 cells with wildtype or mutant human *BMP4* cDNA containing p.Arg198* and p.Glu213* and exposed cells to 0–20 μM PTC124. Treatment with 20 μM PTC124 produced a small, non-significant increase in BMP4 when targeting the p.Arg198* allele, but not the p.Glu213* allele, as measured with an In-cell ELISA assay. We then examined the ability of PTC124 to rescue the ventral tail fin defects associated with homozygosity for the p.Glu209* allele of *bmp4* (*bmp4*^*st72/st72*^) in *Danio rerio*. We in-crossed bmp4^st72/+^ heterozygous fish and found a statistically significant increase in homozygous larvae without tail fin and ventroposterior defects, consistent with phenotypic rescue, after treatment of dechorionated larvae with 0.5 μM PTC124. We conclude that treatment with PTC124 can rescue *bmp4* nonsense variants, but that the degree of rescue may depend on sequence specific factors and the amount of RNA transcript available for rescue. Our work also confirms that zebrafish show promise as a useful animal model for assessing the efficacy of PTC124 treatment on nonsense variants.

## Introduction

For patients with birth defects, pharmacological treatments that impact morphology are not readily available. However, one emerging strategy, termed nonsense suppression therapy (NST), has been considered to hold promise. Approximately 12% of all disease-causing mutations in patients result from premature termination codons (PTCs) that lead to truncated proteins and nonsense-mediated decay of mRNA, causing haploinsufficiency and loss of function [1]. NST utilizes the ability of compounds, such as 3- [5-(2-Fluorophenyl)-1,2,4-oxadiazol-3-yl] benzoic acid (PTC124, also known as Ataluren), to induce translational read-through of nonsense mutations to produce full-length transcripts and prevent premature protein truncation. PTC124 treatment functions by promoting the insertion of a near cognate, aminoacyl tRNAs that results in inclusion of an amino acid, rather than a stop codon, at the ribosomal level [2]. Treatment with PTC124 has been shown to increase full-length, functionally active dystrophin in primary muscle cells from humans and mice that have nonsense mutations in dystrophin and can result in clinical improvement [3–5]. PTC124 has successfully induced *in vivo* suppression of the hCFTR-p.Gly542* nonsense mutation in a mouse model of cystic fibrosis [6] and has corrected nonsense mutations affecting *BMPR2* and *SMAD9* in lung- and blood-derived cells derived from patients with pulmonary arterial hypertension [7]. PTC124 demonstrates reduced toxicity compared to gentamicin and other NST compounds [8] and has been shown to be safe for healthy adult volunteers in single or multiple doses [9]. However, some trials have shown minimal effects from PTC124 treatment for systemic disease [10–12].

The applicability of NST to the treatment of birth defects is only just starting to be researched [13–16]. PTC124 was able to ameliorate the eye defects in a *Pax6*-deficient, murine model of aniridia, even when PTC124 was administered starting at postnatal day 4 (P4), rather than during early eye morphogenesis [14]. In that study, topical administration of PTC124 to the eyes of the transgenic, *Pax6*^Sey +/−^ mice carrying the p.Gly194* allele reversed corneal, lens and retinal malformations, and restored electrophysiological function in the retina [14]. The paper was the first to demonstrate that the postnatal eye retains significant developmental plasticity, with the potential for molecular remodeling after birth. This research led to a clinical trial of PTC124 in patients with aniridia (https://clinicaltrials.gov/ct2/show/NCT02647359). In a more recent study, treatment of postnatal *Pax6*^Sey/+^ mice with ocular defects with PTC124 from the time of spontaneous eye opening at P14 to P60 demonstrated that 1% PTC124 administered in a topical preparation can result in quantitative and stable improvements in eye morphology, histopathology, electroretinograms and behavioral testing [15].

We chose to study the applicability of NST and PTC124 to birth defects. The goal of our study was to determine if NST with PTC124 could rescue or partially rescue the phenotype resulting from nonsense variants in a different developmental gene to *PAX6*, as data on the genes and variants for which NST and PTC124 is effective are still lacking. We selected Bone Morphogenetic Protein 4 (*BMP4*) for this research, as it is highly conserved and expressed early in postnatal development in humans and animal models [17,18]. Deleterious variants in *BMP4* cause a variety of birth defects in humans, including eye defects (anophthalmia, microphthalmia, anterior segment defects, sclerocornea and glaucoma), dental agenesis, diaphragmatic hernia, hydrocephalus, postaxial polydactyly, growth delays and developmental delays [19,20]. In addition, *bmp4*^*st72*/+^ mutant fish that have a nonsense variant, c.625G>T, predicting p.(Glu209*) [21] are available for study. The following work describes our results testing the ability of PTC124 to rescue nonsense variants in *BMP4* cDNA in 293T/17 cells *in vitro* and in the *bmp4*^*s72/+*^ allele in zebrafish *in vivo*.

## Materials and methods

### PTC124 treatment and rescue assayed by Western blotting *in vitro*

293T/17 cells were obtained from the University of California, San Francisco (UCSF) cell culture facility and grown in 10% fetal bovine serum with 0.11 mg/ml Penicillin and Streptomycin. We obtained a full-length, wildtype cDNA clone for *BMP4* inserted into a DDK-tagged expression vector (Cat. No. RC204473, Origene TrueClone Human Collection; Rockville, MD) and used site-directed mutagenesis (Phusion Site-Directed Mutagenesis Kit, Thermo Scientific, Rockford, IL) to generate nonsense mutations in wildtype *BMP4* cDNA. We used Sanger sequencing to verify that the correct sequence variants (c.592C>T, predicting p.(Arg198*) and c.637G>T, predicting p.(Glu213*); NM_001202.3, S1 Table) were present and in the correct reading frame. Cells were grown to 50–70% confluence in 6 well dishes. Cells were then transiently transfected with Lipofectamine 3000 (Life Technologies, Grand Island, NY) diluted in OPTI-MEM and 1–2 μg of empty vector, wildtype *BMP4* cDNA or *BMP4* cDNA containing an in-frame, stop mutation [p.(Arg198*) or p.(Glu213*)]. At 48 hours after transfection, the baseline expression of protein in untransfected and transfected cells was determined using Western blotting. Cell lysates were prepared using RIPA Lysis and Extraction buffer (Thermo Scientific, Rockford, IL) and protein concentrations were measured using the Pierce BCA Protein Assay Kit (Thermo Scientific, Rockford, IL). 15–30μg of total protein was boiled at 70°C for 10 minutes, run on a Novex gel (10% Bis-Tris Plus; Life Technologies, Grand Island, NY) and transferred to a polyvinylidene difluoride (PVDF) membrane using standard methods. We used anti-DDK (SKU TA150030, Origene; Rockville, MD) conjugated to horseradish peroxidase (HRP) at a dilution of 1:1,000 and an ECL chemiluminescence kit for detection (Thermo Scientific, Rockford, IL). We used Glyceraldehyde 3-phosphate dehydrogenase (*GAPDH*) antibody (#3683, Cell Signaling Technology, Danvers, MA) at a dilution of 1:1,000 conjugated to HRP as a loading control. Blots were imaged using the Bio-Rad ChemiDoc Touch Imaging System (Bio-rad laboratories, Hercules, CA).

### PTC124 treatment and rescue assayed by In-cell ELISA assay

PTC124 was obtained from Selleck Chemicals LLC (S6003; Houston TX) and aliquoted as a 10mM stock in dimethylsulfoxide (DMSO). The concentrations and incubation time for PTC124 were determined from previously published dose response curves for PTC124 [22]. At 4 hours after transfection with either empty vector, wildtype *BMP4* cDNA or *BMP4* cDNA containing a nonsense mutation, the medium was replaced with fresh culture medium containing 0 μM, 10 μM or 20 μM PTC124 dissolved in 0.1% DMSO [23]. At 48 hours after addition of PTC124 in DMSO or DMSO only, protein levels were evaluated with the Colorimetric In-Cell ELISA Kit (Pierce Biotechnology, Rockford, IL). Absorbance was measured at 450 nm within 30 minutes of stopping the ELISA reaction. The average 450 nm optical density (OD) for the empty vector wells was determined for each PTC124 dose and subtracted from each well with wildtype or mutant constructs. Expression in the empty vector wells was then normalized to 0 and the results from each data point were plotted without scaling. Error bars were expressed as standard error of the mean. We performed a minimum of five replicate wells for each experimental variable and undertook three independent experiments for each data point. We compared the mean from two independent transfection experiments assayed after 48 hours of PTC124 treatment in 293T/17 cells.

### PTC124 treatment and rescue of *bmp4*^st72/st72^ *in vivo*

*bmp4*^*st72/+*^ fish were obtained from the Zebrafish International Resource Center (ZIRC; Eugene, OR) and wildtype EKW zebrafish were available from the zebrafish core facility at the Cardiovascular Research Institute at the University of California, San Francisco (UCSF). Fish were maintained and bred under standard conditions at 28°C and embryos were staged according to days post fertilization (dpf). Larvae were sacrificed in accordance with the guidelines from the Institute of Animal Care and Use Committee at UCSF using a chemical method followed by a physical method. Larvae that are homozygous for the c.625>T, p.(Glu209*) stop variant in *bmp4* (*bmp4*^*st72/*st72^ larvae) exhibit defects in ventroposterior development, with variable reductions in the development of the ventral tail fin and cloacal defects that can be scored by light microscopy [21]. We examined the ability of PTC124 treatment to rescue the ventral tail fin defects in homozygous, *bmp4*^*st72/st72*^ *Danio rerio* larvae *in vivo* by in-crossing adult fish that were heterozygous for the *bmp4*^*st72*^ allele and treating the resultant eggs/larvae with PTC124 *in vivo*.

### PTC124 treatment and rescue of *bmp4*^st72/st72^ *in vivo*–experiments without dechorionation

In our first group of experiments, *bmp4*^*st72/+*^ heterozygous male and female zebrafish were in-crossed and the eggs were immediately added to plates containing 0 μM, 1 μM, 2.0 μM, 5.0 μM or 10 μM PTC124 dissolved in 0.1% DMSO in E3 medium. Each experimental plate received the same number of eggs from an individual clutch, so that any variation attributable to a specific clutch was minimized. Larvae were scored for phenotypic defects using light microscopy at 3 dpf so that the ventroposterior defects could be scored after tail fin development. We adapted the phenotypic scoring scale developed for *bmp4*^st72^ homozygotes [21] and scored ventroposterior defects as: (i) wildtype (scored as E), (ii) mild reduction/deformity in ventral tail fin (scored as D+ or D), or (iii) severe reduction/deformity in ventral tail fin (scored as C). Fish were also examined for non-specific toxicity, such as generalized edema, cardiac edema, small eye or body size and increased body and tail curvature. After scoring, DNA was extracted from the larvae in 96-well plates by lysis in 1X ThermoPol buffer (New England Biolabs, Ipswich, MA) with 1 μg/μl proteinase K (New England Biolabs, Ipswich, MA) and the larvae were genotyped using Sanger sequencing using primers bmp4_F: TGGTTTGCATCGGATAAAC and bmp4_R: CACATACAGCGCATGTCTCC. We performed three independent experiments. We performed statistics on the genotypes of the treated and untreated, incrossed larvae using Chi-squared analyses and compared the effects of PTC124 treatment on the probability of tail fin defects within the homozygous group of untreated and treated larvae using the Cochran-Armitage test for trend [24].

### PTC124 treatment and rescue of *bmp4*^st72/st72^ *in vivo*–experiments with dechorionation

We repeated the above experiments with dechorionation of larvae at 6–8 hours post fertilization (hpf) by pronase treatment or manual dechorionation prior to the addition of PTC124 in order increase the penetration of the drug. Untreated eggs from *bmp4*^*st72/+*^ fish were incubated in a 1:5 dilution of pronase at 10mg/ml for 1.5 minutes and washed with E3 medium three times. Eggs were then added to petri dishes prepared with 0 μM (control), 0.25 μM and 0.5 μM PTC124 dissolved in 0.1% DMSO in 50ml of E3 medium. The phenotypes were scored at 3 dpf and the larvae were then genotyped with the same methods described as for the non-dechorionated larvae. We performed statistics on the genotypes of the treated and untreated, incrossed larvae using Chi-squared analyses and compared the effects of PTC124 treatment on the probability of tail fin defects within the homozygous group of untreated and treated larvae using the Cochran-Armitage test for trend [24].

### Quantitative reverse transcriptase-polymerase chain reaction (qRT-PCR) to measure *bmp4* expression

We used qRT-PCR to examine the effect of PTC124 treatment on *bmp4* expression. We obtained total RNA at 3 dpf (Zymo Research Quick-RNA Miniprep kit) from 0 μM (control), 0.25 μM, and 0.5 μM of PTC124 treated larvae from the experiments with dechorionation. The RNA was treated with DNase and cDNA was synthesized with the SuperScript III First-Strand Synthesis System (Invitrogen). 1 ng cDNA was amplified using Universal SybrGreen mastermix containing Rox (Roche) and gene specific primers at final concentrations of 2.5 μM. Reactions were run on a StepOne cycler (Thermo Fischer Scientific) and analyzed with StepOne Software and ExpressionSuite software (Thermo Fisher Scientific) according to the ΔΔCt method. We used *eef1a1l1* as an internal control gene. All experiments were performed on three biological replicates from PTC124 treated larvae, and each reaction was run in triplicate. We also performed RT-PCR on cDNA samples obtained from wildtype, EKW larvae and homozygous, *bmp4*^st72/st72^ larvae to determine if *bmp4* was expressed at 6 hpf and 1 dpf using primers *eef1a1l1*_Lim_F: CCAACTTCAACGCTCAGGTCA *eef1a1l1*_Lim_R: CAAACTTGCAGGCGATGTGA to amplify a 105 basepair fragment and primers *bmp4*_qRT PCR_F: CGCCTCTGCGATTCGTTTTTA and *bmp4*_qRT PCR_R CCCCTGTTTGATCTGGGTCTG to amplify a 123 basepair fragment.

## Results

### PTC124 treatment and rescue assayed by Western blotting *in vitro*

We generated two nonsense variants in full length, human *BMP4* cDNA for the *in vitro* experiments. The first stop variant, c.592C>T, predicting p.(Arg198*) (NM_001202.3; S1 Fig), was detected in a patient with anophthalmia, microphthalmia, sclerocornea, diaphragmatic hernia and hydrocephalus [19]. The second variant, c.637G>T, predicting p.(Glu213*) (NM_001202.3; S1 Fig), is the human orthologue of the c.625>T, p.(Glu209*) mutation (NM_131342) that is present in the *bmp4*^*st72/+*^ zebrafish line [21]. Although this variant is not known to be associated with a phenotype in humans, homozygosity for the *bmp4*^*st72/+*^ allele was associated with ventroposterior defects and cloacal defects in *Danio rerio* [21]. As alignment of both the human and the zebrafish proteins shows that the BMP4 protein is highly conserved (S1 Fig), both of these nonsense variants are likely to be functionally important. After transfection of 293T/17 cells with full length wildtype *BMP4* cDNA, cDNA containing p.(Arg198*) and cDNA containing p.(Glu213*), Western blotting with an anti-DDK antibody showed a robust band at the expected site for BMP4 (S2 Fig, lane 7), but there was minimal detectable protein after transfection of cDNA containing p.(Arg198*) (lane 3) and almost no detectable protein following transfection of cDNA containing p.(Glu213*) (lane 5). The Western blot thus confirmed the anticipated reduction in stable BMP4 protein associated with the nonsense variants p.(Arg198*) and p.(Glu213*). Western blotting did not detect rescue after treatment of the cells with 10 μM PTC124 for 48 hours (S2 Fig, lanes 4 and 6).

### PTC124 treatment and rescue assayed by In-cell ELISA assay

**Fig 1:**
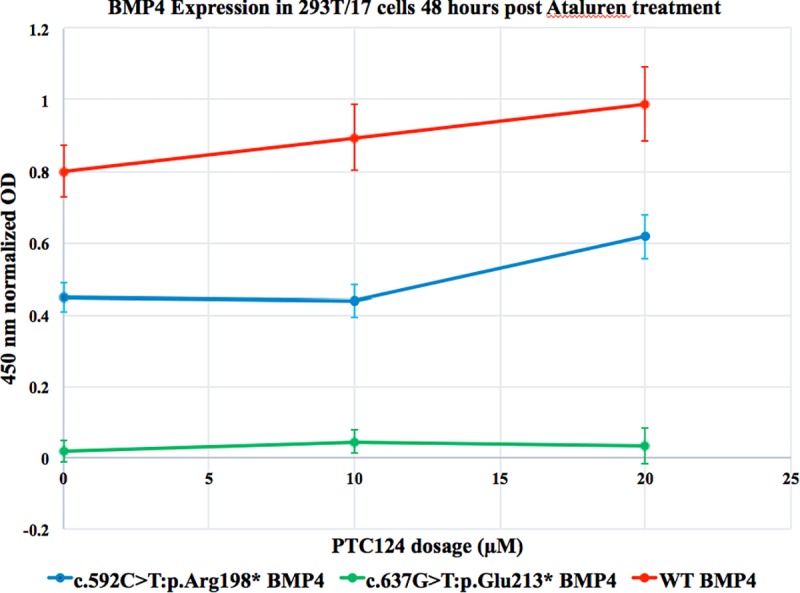
In-cell ELISA assay to assess the ability of PTC124 to rescue the p.(Arg198*) and p.(Glu213*) variants in *BMP4* in 293T/17 cells. Results of In-cell ELISA assay, demonstrating BMP4 protein levels for the p.(Arg198*) and p.(Glu213*) variants after incubation of 293T/17 cells for 48 hours with 20 μM PTC124, compared to untreated cells transfected with the same construct. An increase in BMP4 protein level was seen with p.(Arg198*), but this was not significant (p value = 0.09). There was minimal increase in the construct containing p.(Glu213*) after treatment with 10 μM PTC124 and 20 μM PTC124.

We subsequently used an In-cell ELISA assay to measure the amount of protein after PTC124 treatment *in vitro*, as this assay is considered to be more sensitive for the detection of protein levels compared to Western blotting [23]. We found that the expression of p.(Arg198*) increased after treatment with 20 μM PTC124, although the increase in expression was not statistically significant (p = 0.09; Fig 1, In-cell ELISA assay to assess the ability of PTC124 to rescue the p.(Arg198*) and p.(Glu213*) variants in *BMP4* in 293T/17 cells, and S2 Table). There was no change in expression of p.(Glu213*) (Fig 1 and S2 Table). Expression of wildtype BMP4 increased after PTC124 treatment, although the increase was less than for the p.(Arg198*) variant and was not statistically significant (S2 Table). We did not observe any changes in protein level for untransfected cells and cells incubated in DMSO without PTC124. We did not attempt concentrations higher than 20 μM PTC124 due to the toxicity that has been observed *in vitro* with PTC124 treatment at 40 μM [23].

### PTC124 treatment and rescue of *bmp4*^st72/st72^ *in vivo*

**Fig 2:**
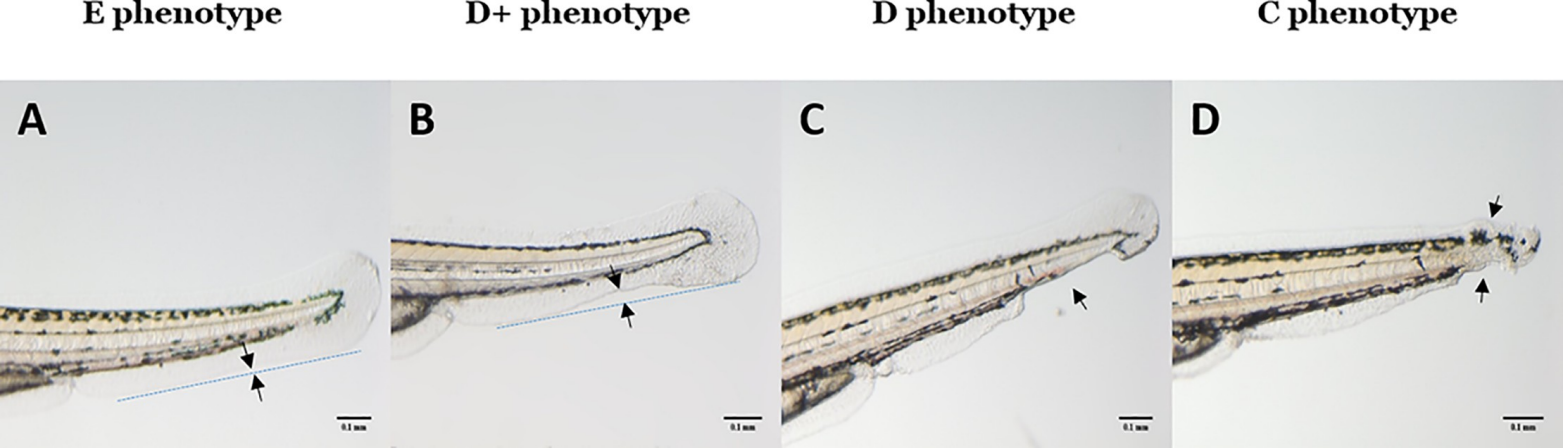
Ventroposterior and tail fin defects in *bmp4*^*st72/st72*^ homozygous larvae at 3 days post fertilization. Representative examples of ventroposterior defects scored in *bmp4*^*st72/st72*^ homozygous larvae at 3 days post fertilization. The panels (Fig 2A–2D) show the tail fin (scale bars = 100 μm). Fig 2A shows a larva scored as an E phenotype (typical appearance with no defects), Fig 2B and 2C show larvae scored as a D phenotype (mild and moderate tail fin defects) and Fig 2D shows a larva scored as a C phenotype (moderate to severe tail fin defect). The sites of the tail fin defects are marked with arrows, except for Fig 2A, in which the arrows show a wildtype tail fin.

Representative examples of the tail fin defects scored in larvae homozygous for the *bmp4*^*st72*^ allele are shown in Fig 2, Ventroposterior and tail fin defects in *bmp4*^*st72/st72*^ homozygous larvae at 3 days post fertilization. Our phenotypic analysis of in-crossed, *bmp4*^*st72/+*^ heterozygous *Danio rerio* confirmed the range of phenotypic defects observed in homozygotes [21], with mild to severe reduction in development of the ventral tail fin and rare cloacal defects. Tail fin defects were observed at all concentrations of PTC124 tested, but the penetrance of the tail fin defects in *bmp4*^*st72/*st72^ homozygous larvae varied, ranging from 22% to 79% without PTC124 treatment. These findings are similar to the range of dorsalization defects (8.6–92%) observed by Stickney et al. [21]. As the penetrance of the tail fin defects was a variable that could influence our assessment of rescue, we analyzed only experiments where the penetrance of the tail fin defects was above 50% in homozygotes without PTC124 treatment.

When all phenotypic categories (E, D and C) are combined for each experiment, the segregation of the st72 allele is consistent with Mendelian inheritance (Tables 1 and 2).

**Table 1:** Phenotypic and genotypic analysis at 72 hours post fertilization (hpf) following treatment of in-crossed heterozygous *bmp4*^*st72/+*^ zebrafish with 0 μM, 1.0 μM and 2.0 μM PTC124. Larvae were not dechorionated and PTC124 treatment was started at 0 hpf. Results are presented as the total of 3 (0 μM, 1.0 μM) or 2 (2.0 μM) independent experiments.

PTC124 dose	Phenotype	Wildtype	Heterozygous	Homozygous	Total	Chi square (2 d.f.)^a^	p-value
	C	0	0	3	3	9	0.011
	D or D+	1	8	31	40	59.4	<0.001
0 μM	E	32	69	16	117	8.145	0.017
	Total	33	77	50	160	3.838	0.147
	C	0	0	0	0	-	-
1 μM	D or D+	2	5	31	38	64.895	<0.001
	E	45	85	17	147	14.265	<0.001
	Total	47	90	48	185	0.146	0.93
	C	0	1	0	1	1	0.607
2 μM^b^	D or D+	1	3	13	17	24.059	<0.001
	E	25	45	9	79	8.013	0.018
	Total	26	49	22	97	0.34	0.844

Chi-squared^a^ analysis was performed to compare observed from expected values for autosomal recessive inheritance with 2 degrees of freedom. 2.0 μM^b^–only two experiments were performed at this dose level

**Table 2:** Phenotypic and genotypic analysis at 72 hours post fertilization (hpf) following treatment of incrossed heterozygous *bmp4*^*st72/+*^ zebrafish with 0 μM, 0.25 μM and 0.5 μM PTC124. Larvae were dechorionated at 6–8 hpf prior to starting PTC124 treatment. Results are presented as the total of 3 independent experiments for all PTC124 doses.

PTC124 dose	Phenotype	Wildtype	Heterozygous	Homozygous	Total	Chi square (2 d.f.)^a^	p-value
	C	0	0	3	3	9	0.011
	D or D+	1	12	27	40	40.2	<0.001
0 μM	E	31	68	7	106	19.36	<0.001
	Total	32	80	37	149	1.15	0.568
	C	0	0	0	0	-	-
0.25 μM	D or D+	1	3	20	24	43.58	<0.001
	E	33	64	7	104	18.54	<0.001
	Total	34	67	27	128	1.05	0.592
	C	0	0	0	0	-	-
0.5 μM	D or D+	1	6	12	19	15.32	<0.001
	E	28	69	20	117	4.863	0.088
	Total	29	75	32	136	1.57	0.455

Chi-squared^a^ analysis was performed to compare observed from expected values for autosomal recessive inheritance with 2 degrees of freedom.

**Fig 3:**
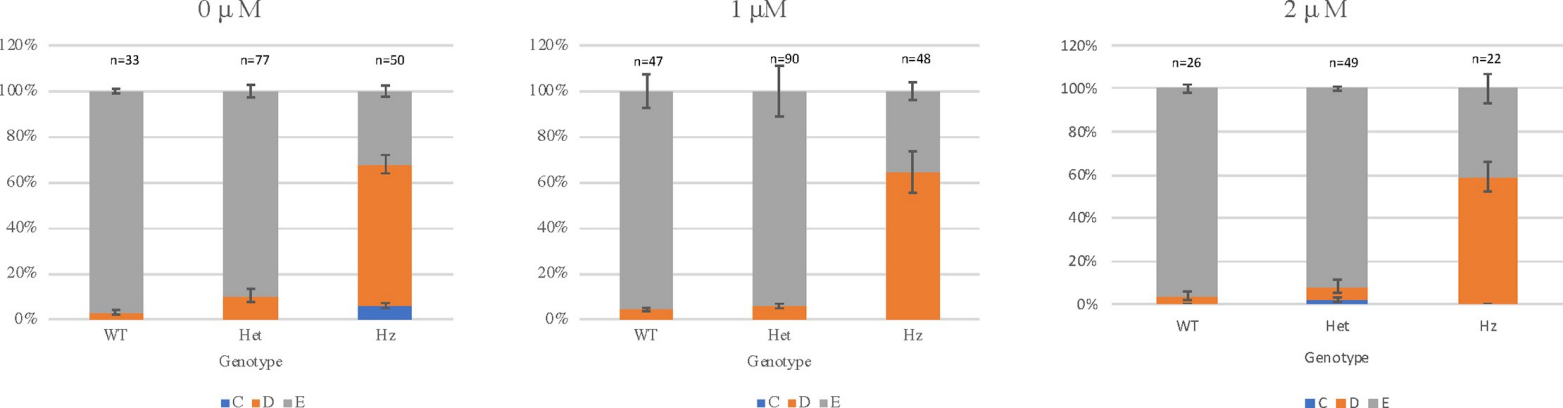
Results of phenotypic and genotypic analysis following treatment of in-crossed heterozygous *bmp4*^*st72/+*^ zebrafish with 0 μM, 1 μM and 2 μM PTC124. Larvae were not dechorionated and PTC124 treatment was started at 0 hours post fertilization (hpf). Analysis was performed at 72 hpf and the results are shown as the mean of 3 independent experiments, except for treatment with 2 μM PTC124, which is shown as the mean of 2 independent experiments. Larvae were scored as wildtype (E), mild reduction/deformity in ventral tail fin (D+ or D) or severe reduction/deformity in ventral tail fin (C). WT = wildtype, het = heterozygote, HZ = homozygote. Error bars show standard error of the mean. We tested for trend in the probability of tail fin defects across the increasing doses of 0 μM, 1.0 μM and 2.0 μM PTC124 treated, groups of homozygous larvae [24]. The probability of tail fin defect decreased from 68% to 64.58% and 59.09% as the dose increased to 1.0 μM and 2.0 μM. However, the trend failed to meet the statistical significance (p = 0.4681).

However, when the phenotypic categories are considered separately in untreated larvae, the ventral tail fin defects (D and C) are seen more frequently with homozygous genotypes. At 0 μM PTC124, the D phenotype, with mild to moderate tail fin defects, is seen significantly more frequently in homozygous larvae compared to wildtype or heterozygous larvae (p<0.001), in accordance with the expected, autosomal recessive inheritance of the *bmp4*^*st72*^ allele and its association with tail fin defects (Tables 1 and 2). Similarly, the E phenotype (wildtype) is significantly more frequent in larvae with wildtype genotypes for 0 μM PTC124 treatment (p = 0.017 for undechorionated larvae, Table 1 and Fig 3, Results of phenotypic and genotypic analysis following treatment of in-crossed heterozygous *bmp4*^*st72/+*^ zebrafish with 0 μM, 1 μM and 2 μM PTC124).

For treated larvae, we reasoned that if PTC124 could rescue the phenotype, this would manifest as an increase in non-penetrance for the ventroposterior fin defects for larvae with a homozygous, *bmp4*^*st72/st72*^ genotype (i.e. more larvae with a homozygous genotype would appear phenotypically wildtype). In the experiments without dechorionation, treatment with 1 μM and 2 μM PTC124 from 0 hpf did not significantly change the penetrance of the ventroposterior fin defects compared to untreated larvae (Table 1, Fig 3) and thus there was no evidence of phenotypic rescue. Toxicity was also observed after treatment with 1 μM and 2 μM PTC124 (S3 Table), with a mean of 28.5% larvae demonstrating abnormalities comprising a curved body axis, generalized edema, cardiac edema and microphthalmia after 2 μM PTC124 treatment, compared to an average of 5.6% of larvae with toxicity at 0 μM PTC124 (S3 Table). Higher concentrations of PTC124 (5 μM and 10 μM PTC124) were also used in preliminary experiments and resulted in at least 50% larvae manifesting non-specific toxicity and these concentrations of PTC124 were not further evaluated. These findings are consistent with previously observed negative effects at higher PTC124 concentrations (5 μM and 35 μM) when tested in dystrophin-null, Sapje zebrafish [25].

**Fig 4:**
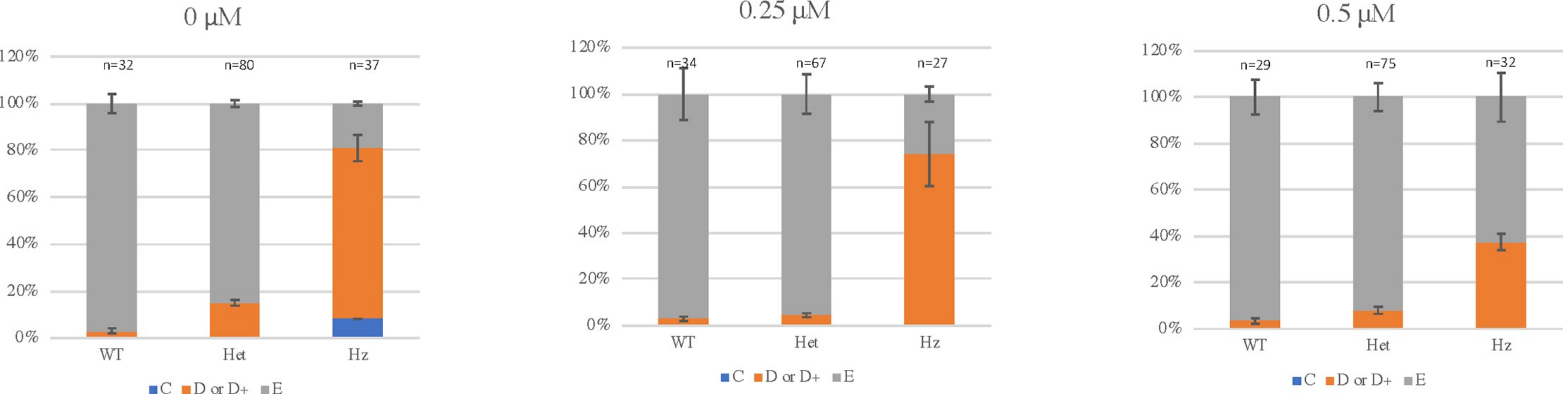
Phenotypic and genotypic analysis following treatment of incrossed heterozygous *bmp4*^*st72/+*^ zebrafish with 0 μM, 0.25 μM and 0.5 μM PTC124. Larvae were dechorionated at 6–8 hours post fertilization (hpf) prior to starting PTC124 treatment. Analysis was performed at 72 hpf and the results are shown as the mean of 3 independent experiments. Larvae were scored as wildtype (E), mild reduction/deformity in ventral tail fin (D+ or D) or severe reduction/deformity in ventral tail fin (C). WT = wildtype, het = heterozygote, HZ = homozygote. Error bars show standard error of the mean. We tested for trend in the probability of tail fin defects across the increasing doses of 0 μM, 0.25 μM and 0.5 μM PTC124 treated, groups of homozygous larvae [24]. The probability of tail fin defect decreased from 81.08% to 74.07% and 37.50% as the dose increased to 0.25 μM and 0.5 μM. The decreasing trend was statistically significant (p = 0.0002).

**Fig 5:**
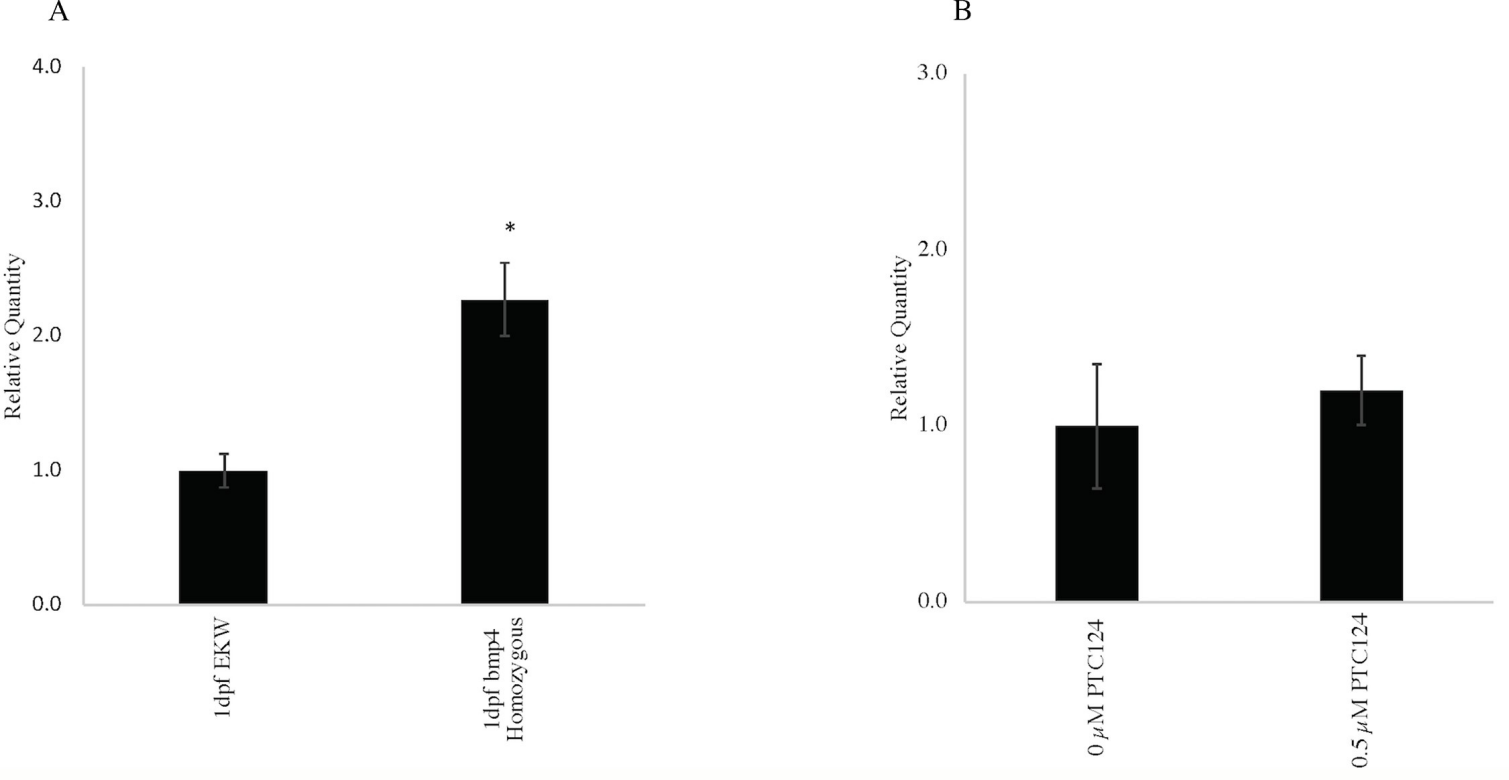
Quantitative reverse transcriptase-polymerase chain reaction to assess *bmp4* expression. Fig 5A. Quantitative reverse transcriptase-polymerase chain reaction (qRT-PCR) showing *bmp4* expression in untreated wildtype and *bmp4*^*st72/st72*^ homozygous larvae at 1 day post fertilization (dpf). RNA was extracted at 1 dpf from untreated, pooled wildtype EKW larvae and *bmp4*^*st72/st72*^ homozygous larvae. After cDNA synthesis, qRT-PCR was performed and analyzed using the ΔΔCt method and *eef1a1l1* as an internal control gene. The results, plotted as relative quantity (RQ) normalized to *bmp4* expression in wildtype EKW larvae at 1.0 show increased *bmp4* expression in *bmp4*^*st72/st72*^ homozygous larvae (p = 0.014), consistent with *bmp4* RNA available for rescue using nonsense suppression therapy. All values are an average of 3 replicates and 3 biological replicates were performed for each experiment. The error bars indicate the standard error (SE) of the mean. Fig 5B. Quantitative reverse transcriptase-polymerase chain reaction (qRT-PCR) showing *bmp4* expression in incrossed *bmp4*^*st72/+*^ heterozygous larvae treated with 0 μM PTC124 and incrossed *bmp4*^*st72/+*^ heterozygous larvae treated with 0.5 μM PTC124 from 6–8 hours post fertilization (hpf). RNA was extracted at 3 days post fertilization (dpf) from pooled larvae treated with 0 μM PTC124 and larvae that had been treated with 0.5 μM PTC124 from 6–8 hpf. After cDNA synthesis, qRT-PCR was performed and analyzed using the ΔΔCt method and *eef1a1l1* as an internal control gene. The results, plotted as relative quantity (RQ) normalized to *bmp4* expression (1.0) in incrossed, *bmp4*^*st72/+*^ heterozygous larvae that were treated with 0 μM PTC124 show a non-significant increase in *bmp4* expression in incrossed, *bmp4*^*st72/+*^ heterozygous larvae treated with 0.5 μM PTC124 (p = 0.09). All values are an average of 3 replicates and 3 biological replicates were performed for each experiment. The error bars indicate the standard error (SE) of the mean.

For the experiments with dechorionation at 6 to 8 hpf and commencement of PTC124 treatment after dechorionation, lower doses of PTC124 were chosen for evaluation, as 1 μM and 2 μM PTC124 produced unacceptable toxicity, implying a degree of non-penetrance of the medication through the chorion. In the larvae treated with 0.5 μM PTC124, an increase in non-penetrance of the ventroposterior fin defects was observed in homozygous larvae compared to untreated larvae (Table 2; Fig 4, Phenotypic and genotypic analysis at 72 hours post fertilization following treatment of incrossed heterozygous *bmp4*^*st72/+*^ zebrafish with 0 μM, 0.25 μM and 0.5 μM PTC124). The probability of a tail fin defect in a larva with a homozygous genotype decreased from 81.08% to 74.07% and 37.50% as the dose increased from 0 μM to 0.25 μM and 0.5 μM (Table 2). The decreasing trend was statistically significant (p = 0.0002). The result for 0.5 μM PTC124 is thus consistent with rescue of the phenotype by PTC124. We therefore attempted to determine if PTC124 treatment would result in a difference in the expression of *bmp4* mRNA using qRT-PCR and *bmp4* specific primers. We did observe *bmp4* transcript at 6 hpf and 1 dpf in homozygous larvae and EKW wildtype larvae as we could amplify the cDNA using gene specific primers, confirming that transcript was available for rescue (Fig 5A Quantitative reverse transcription-polymerase chain reaction to assess *bmp4* expression in *bmp4*^*st72/st72*^ homozygous larvae at 1 day post fertilization, and S3 Fig Quantitative reverse transcription-polymerase chain reaction showing *bmp4* transcript at 6 and 24 hours post fertilization in wildtype EKW and homozygous *bmp4*^st72/st72^ larvae). Our results confirmed a modest increase in *bmp4* RNA after treatment with 0.5 μM PTC124, but this increase was not statistically significant (Fig 5B Quantitative reverse transcriptase-polymerase chain reaction to assess *bmp4* expression in 0 μM and 0.5 μM PTC124 treated larvae at 3 days post fertilization).

In the experiments with dechorionation, the toxicity for untreated and treated larvae was 11.4% for 0 μM, 3.91% for 0.25 μM and 4.41% for 0.5 μM PTC124 (S4 Table). At 1 μM and 2 μM PTC124, toxicities in the dechorionated experiments were higher and we did not study these concentrations. Larvae were unable to be dechorionated earlier than 6 hpf due to toxicity in our hands.

## Discussion

We used PTC124 to attempt NST and rescue of nonsense mutations in *BMP4*, both *in vitro* in 293T/17 cells and *in vivo* in *Danio rerio* that were homozygous for the *bmp4*^*st72/st72*^ allele. For the *in vitro* experiments, PTC124 rescue for various genes has been estimated at 2 to 5.5% [1] and we were anticipating only a 2–13% increase in protein levels after NST based on data from cell lines and animal models [1,22]. This level of rescue is difficult to detect by Western blotting, and as there still exists controversy regarding PTC124 and its ability to bind to luciferase, we decided to use an In-cell ELISA assay to measure protein levels after PTC124 treatment. We found a modest, non-significant increase in BMP4 after transfection of the p.(Arg198*) allele with 20 μM PTC124, but not for transfection of the p.(Glu213*) allele (Fig 1). Rescue after PTC124 treatment has been influenced by the nucleotide sequence of the stop codon (UGA>UAG>UAA) [1,26] and the immediate 3’ nucleotide present in the sequence [23,27]. Interestingly, the increase in BMP4 protein levels after Ataluren treatment of cells with the p.(Arg198*) mutation, which has a UGA stop codon, was greater than for the p.(Glu213*) mutation, which has a UAA stop codon. These findings are consistent with the variation observed with different nucleotide sequences for stop codons as described above [1,2,26]. The response to NST has previously been shown to be variable, even in patients with the same nonsense variant [28]. It is also important to note that the level of PTC-bearing transcripts that are the target for readthrough can vary depending on the variant and other factors and this may limit response, as patients with markedly reduced levels of nonsense transcript show no response to NST, whilst responses can be demonstrated in patients with higher levels of transcript [29]. The degree of rescue can thus be altered by variation in the genes and proteins that participate in NMD and that therefore influence the levels of available transcript for NST. In our experiments, the Western blot performed after transfection of wildtype and variant cDNA without PTC124 treatment demonstrated a greater amount of protein, and thus transcript, for the p.(Arg198*) variant compared to the p.(Glu213*) variant (S2 Fig). The increased efficacy of rescue for the earlier stop codon may thus have been influenced by the greater level of available transcript for rescue. For the *in vivo* experiments, we were able to demonstrate residual transcript available for rescue by breeding homozygous *bmp4*^st72/st72^ fish and performing RNA extraction and qPCR at 6 and 24 hpf (S3 Fig). Other factors associated with variation in successful readthrough include the sequence up- and downstream from the stop codon, the fourth nucleotide immediately after the stop codon [30] and the presence of a cytosine (C) residue after either UGA and UAA stop codons [26,31]. Readthrough can also be influenced by tissue-specific differences [28].

We tested two nonsense mutations in human *BMP4 in vitro* - c.592C>T, predicting p.(Arg198*) and c.637G>T, predicting p.(Glu213*). These mutations remove the transforming growth factor-beta, C-terminal domain and result in truncation of more than 10% of the protein and thus both are predicted to result in loss of function. However, it is unclear if either mutation is associated with nonsense mediated decay (NMD), as they both occur in the ultimate exon of the gene and it is possible that both mutations result in a shortened protein that does not exert a biological effect, but also does not decay. Our anti-DDK tag was added at the C-terminus of the protein, and thus we could not observe shorter or prematurely truncated proteins with this antibody.

For the *in vivo* studies, the *bmp4*^*st72*^ allele causes truncation of bmp4 prior to the mature peptide domain and was predicted to eliminate all function, as *bmp4* mRNA with the st72 p.(Glu209*) nonsense mutation has been shown to have no activity, either ventralizing or dorsalizing, in overexpression assays [21]. As *bmp4* is weakly expressed at 1 and 3 hpf and strongly expressed from 16 hpf to 7 dpf [32,33], our first experiments commenced PTC124 treatment essentially from the time of fertilization, without dechorionation of embryos. One advantage of early treatment is that there is no need to standardize the start of treatment relative to the developmental stages of larvae. However, it is possible that early treatment was the cause of the increased toxicity that we observed in these experiments. In addition, there is little data on the ability of PTC124 to penetrate the chorion and we did not test for PTC124 in the early larvae, although our finding of dose dependent toxicity after treatment suggests that this small compound does penetrate the chorionic membrane. It is possible that we observed toxicity, but not rescue, in these experiments due to increased sensitivity to the compound early in embryogenesis, or global read through of non-mutant alleles that could perturb development. However, other researchers have reported rescue without significant non-specific effects at similar concentrations to the ones that we tested [1].

We therefore performed experiments with addition of PTC124 after dechorionation at 6–8 hpf; we were unable to dechorionate embryos effectively and without toxicity at earlier timepoints. Other studies have also commenced treatment at later timepoints, starting at 72 hpf with Sapje zebrafish [25] and from 10 hpf with dechorionated embryos [34]. The results for our dechorionation experiments did demonstrate an increase in the wildtype phenotype in larvae that were homozygous (*bmp4*^*st72/st72*^), consistent with rescue at 0.5 μM, but there was no increase in wildtype phenotype with 0.25 μM PTC124 treatment (Table 2 and Fig 3). qRT-PCR experiments showed increased *bmp4* mRNA after treatment with 0.5 μM PTC124, but the increase in mRNA was not statistically significant. Our results show that *Danio rerio* may have promise as a model system to assess the effects of PTC124 on specific gene variants and experiments with other zebrafish lines with a less variable phenotype could be attempted in the future.

NST has been successfully trialed using a zebrafish model of human disease. In Sapje zebrafish that have a nonsense mutation in the dystrophin gene, PTC124 can improve the contractile function of skeletal muscle and increase dystrophin expression [25]. Gentamicin was also successfully used as an NST agent to rescue two models of coloboma induced by mutations in the *pax2*.*1* and *lamb1* genes [1]. However, our findings emphasize that the successful application of PTC124 and NST may depend on the specific gene, in terms of timing of gene expression, the specific sequence of the nonsense codon and the availability of nonsense transcript for read-through as described above. Although NST is typically only predicted to result in partial rescue, even a small increase in mRNA or protein may be sufficient to ameliorate the effects of deleterious sequence variants in autosomal recessive disease [35]. For example, after NST directed against mutations in the *USH1C* gene, only 2.5% of harmonin expression was induced [35]. Thus, NST may still have utility as an adjuvant therapy for specific nonsense variants.

In summary, we investigated the ability of PTC124 treatment to rescue the p.(Arg198*) and p.(Glu213*) *BMP4* alleles *in vitro*. Although treatment with 20 μM PTC124 increased BMP4 protein levels for the p.(Arg198*) variant as measured by an In-cell ELISA assay, the increase was not significant and there was minimal effect on p.(Glu213*) protein levels. We then examined the ability of PTC124 treatment to rescue the phenotype associated with the *bmp4*^*st72/st72*^ nonsense allele in zebrafish, using treatments of 0–2 μM from 0 hpf in non-dechorionated larvae and 0–0.5 μM after dechorionation of larvae at 6–8 hpf. There was a statistically significant increase in *bmp4*^st72/st72^ homozygous larvae with a wildtype phenotype after 0.5 μM PTC124 treatment in the dechorionated larvae, consistent with rescue. We conclude that the degree of rescue may depend on sequence specific factors and the amount of RNA transcript available for rescue. Our work also confirms that zebrafish show promise as a useful animal model for assessing the efficacy of PTC124 treatment on nonsense variants.

## Supporting information

S1 Fig Chromatograms showing variants in human *BMP4* cDNA (NM_001202.3) and alignment of *BMP4* with zebrafish *bmp4*. S1A Fig Chromatogram showing c.592C>T, predicting p.(Arg198*) in human *BMP4* cDNA (NM_001202.3). The top tracing is wildtype sequence, the bottom shows the nonsense variant. S1B Fig Chromatogram showing c.637G>T, predicting p.(Glu213*) in human *BMP4* cDNA (NM_001202.3). The top tracing is wildtype sequence, the bottom shows the nonsense variant. S1C Fig Alignment of human BMP4 (BMP4-001) and zebrafish bmp4 (bmp4-001) proteins, showing site of BMP4 variants studied and site of p.(Glu209*) variant, as found with the *bmp4*^*st72*^ allele.
(TIFF)

S2 Fig Western blotting to assess the ability of PTC124 to rescue the p.Arg198* and p.Glu213* variants in *BMP4* in 293T/17 cells. Western blot at 48 hours after transfection of 293T/17 cells with wildtype and BMP4 cDNA containing c.592C>T, predicting p.(Arg198*), and c.637G>T, predicting p.(Glu213*). Detection was accomplished using anti-DDK antibody. Wildtype *BMP4* is strongly expressed at the expected size (approximately 50 kDa) whereas there is negligible expression for either variant cDNA. GAPDH was used as a loading control.
(TIFF)

S3 Fig Reverse transcription-polymerase chain reaction showing *bmp4* transcript at 6 and 24 hours post fertilization in wildtype EKW and homozygous *bmp4*^st72/st72^ larvae. Amplification of *eef1a1l1* in wildtype EKW larvae and homozygous *bmp4*^st72/st72^ larvae at 6 hours post fertilization (hpf) and 1 day post fertilization (dpf) shows the expected 105 basepair band for both genotypes. Amplification of *bmp4* in wildtype EKW larvae and homozygous *bmp4*^st72/st72^ larvae at 6 hpf and 1 dpf also shows the expected 123 basepair band for both genotypes. Control lanes with water rather than cDNA are negative for amplified bands. Each marker band represents 100 basepairs.
(TIFF)

S1 Table Nonsense variants in human *BMP4* and *Danio rerio bmp4*. (PDF)

S2 Table Data from In-cell ELISA assay after treatment of 293T/17 cells with 0–20 μM PTC124. The average 450 nm optical density (OD) for the empty vector wells was determined for each PTC124 dose and subtracted from each well with wildtype or mutant constructs. Expression in the empty vector wells was normalized to 0 and the results from each data point were plotted without scaling.
(PDF)

S3 Table PTC124 treatment at 1 μM or 2 μM increases non-specific toxicity in treated larvae compared to untreated larvae at 72 hours post fertilization (hpf) in *bmp4^st72/+^* incrossed zebrafish. Larvae were not dechorionated and PTC124 treatment was started at 0 hpf. Each measurement is the mean of 3 or 4 independent experiments; data includes results from experiments not included in Table 1 due to low penetrance of ventroposterior defects in control homozygous larvae in the experiment.
(PDF)

S4 Table PTC124 treatment at 0.25 μM or 0.5 μM does not increase non-specific toxicity in dechorionated larvae compared to untreated larvae at 72 hours post fertilization (hpf) in *bmp4^st72/+^* incrossed zebrafish. Larvae were dechorionated at 6–8 hpf and PTC124 treatment was started at 6–8 hpf. Each measurement is the mean of 3 independent experiments.
(PDF)
