# Supplementary figures and images for: Use of PTC124 for nonsense suppression therapy targeting BMP4 nonsense variants in vitro and the bmp4st72 allele in zebrafish
Source: PLoS One. 2019 Apr 24;14(4):e0212121. doi: 10.1371/journal.pone.0212121 (PMC6481805; doi:10.1371/journal.pone.0212121)

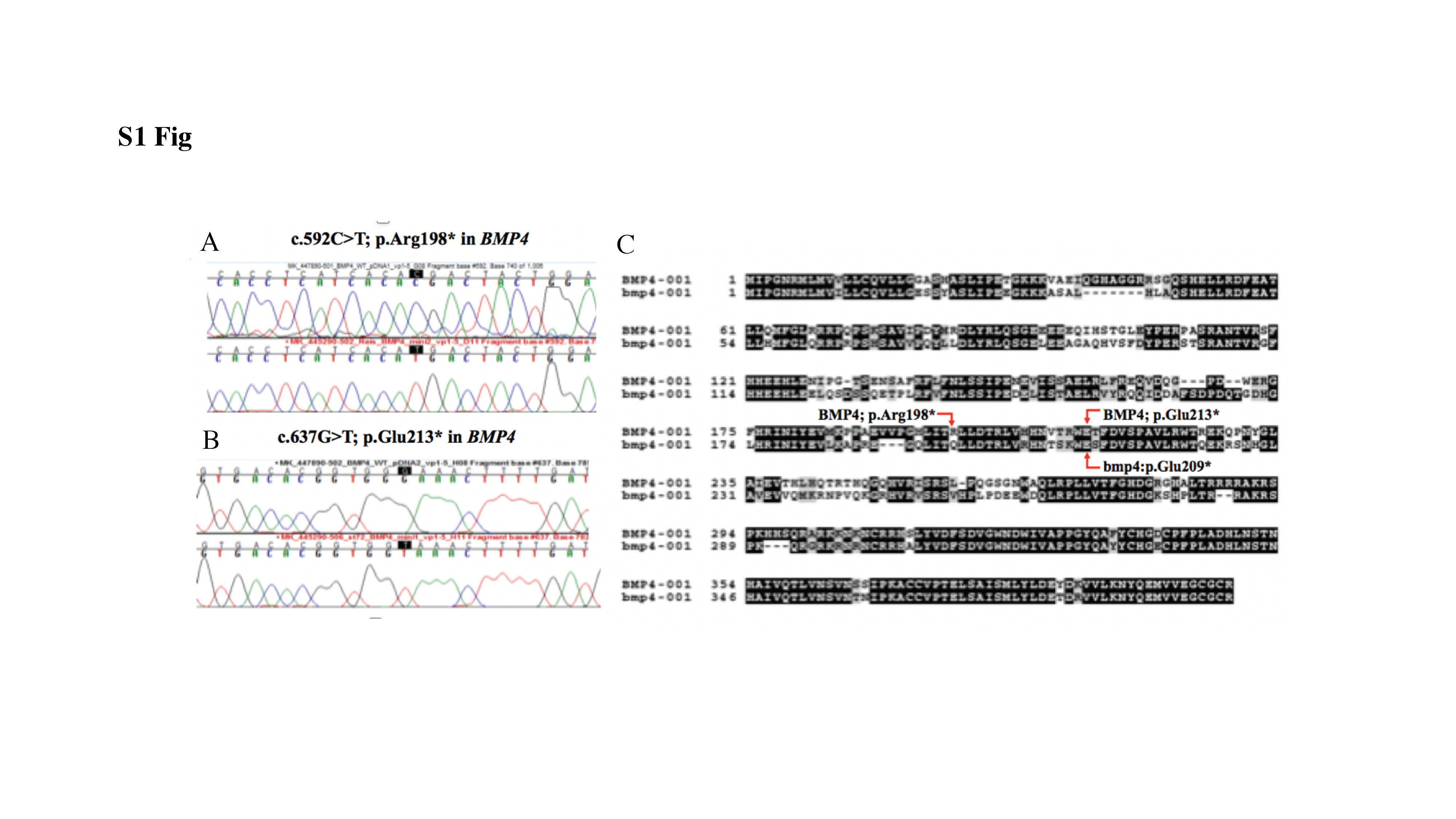

Supplement: S1 Fig — S1A Fig Chromatogram showing c.592C>T, predicting p.(Arg198*) in human BMP4 cDNA (NM_001202.3). The top tracing is wildtype sequence, the bottom shows the nonsense variant. S1B Fig Chromatogram showing c.637G>T, predicting p.(Glu213*) in human BMP4 cDNA (NM_001202.3). The top tracing is wildtype sequence, the bottom shows the nonsense variant. S1C Fig Alignment of human BMP4 (BMP4-001) and zebrafish bmp4 (bmp4-001) proteins, showing site of BMP4 variants studied and site of p.(Glu209*) variant, as found with the bmp4st72 allele. (TIFF) [file pone.0212121.s001.tiff]

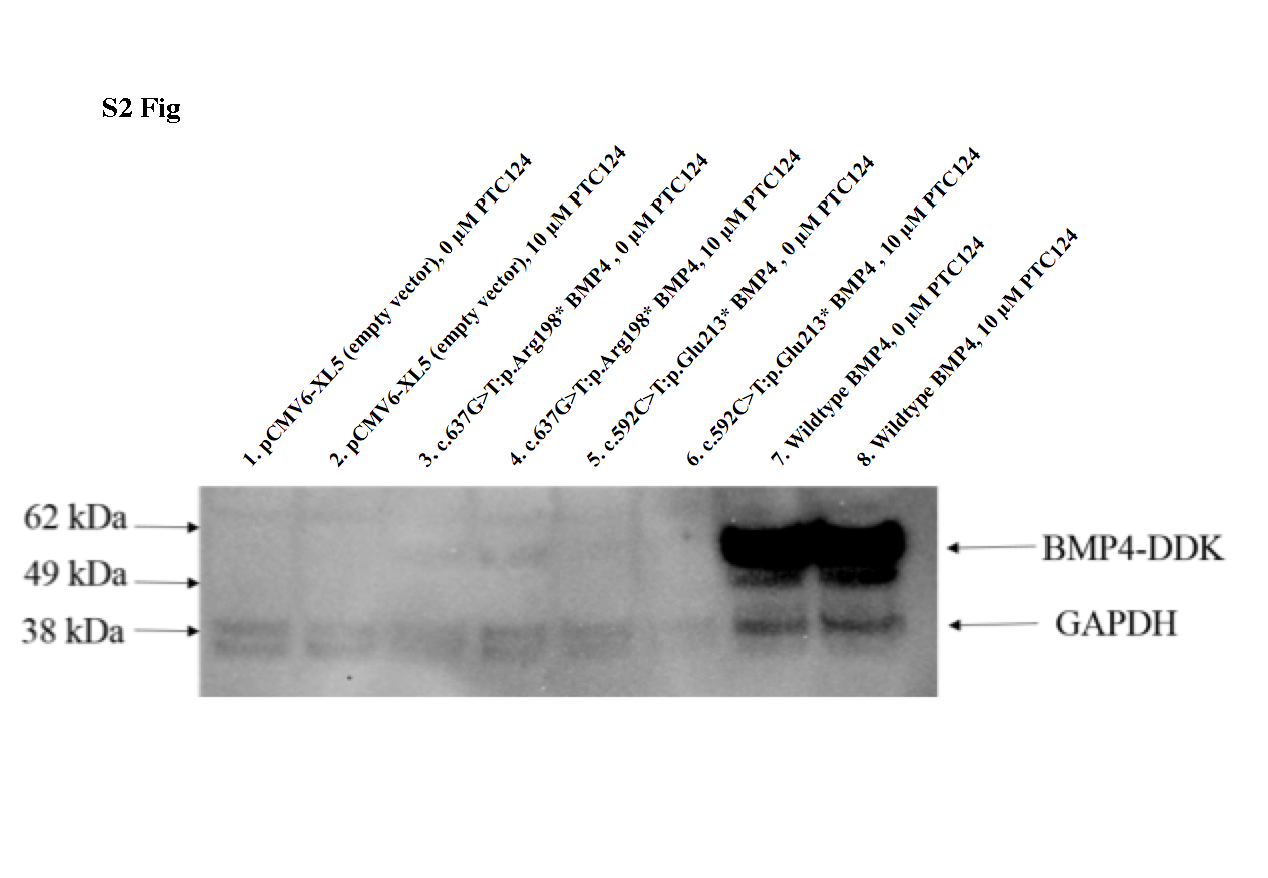

Supplement: S2 Fig — Western blot at 48 hours after transfection of 293T/17 cells with wildtype and BMP4 cDNA containing c.592C>T, predicting p.(Arg198*), and c.637G>T, predicting p.(Glu213*). Detection was accomplished using anti-DDK antibody. Wildtype BMP4 is strongly expressed at the expected size (approximately 50 kDa) whereas there is negligible expression for either variant cDNA. GAPDH was used as a loading control. (TIFF) [file pone.0212121.s002.tiff]

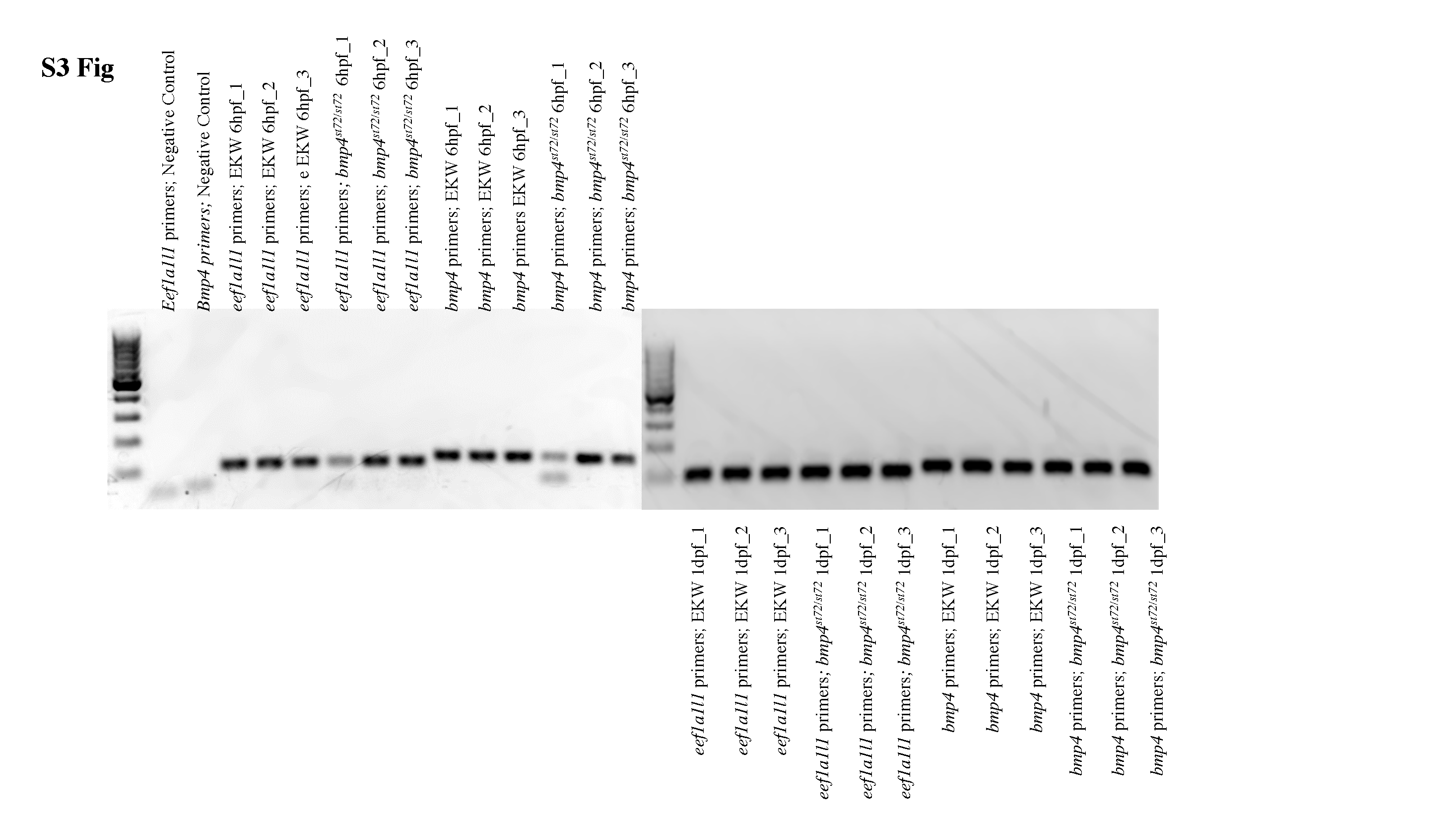

Supplement: S3 Fig — Amplification of eef1a1l1 in wildtype EKW larvae and homozygous bmp4st72/st72 larvae at 6 hours post fertilization (hpf) and 1 day post fertilization (dpf) shows the expected 105 basepair band for both genotypes. Amplification of bmp4 in wildtype EKW larvae and homozygous bmp4st72/st72 larvae at 6 hpf and 1 dpf also shows the expected 123 basepair band for both genotypes. Control lanes with water rather than cDNA are negative for amplified bands. Each marker band represents 100 basepairs. (TIFF) [file pone.0212121.s003.tiff]
