# Supplementary material for: Use of PTC124 for nonsense suppression therapy targeting BMP4 nonsense variants in vitro and the bmp4st72 allele in zebrafish
Source: PLoS One. 2019 Apr 24;14(4):e0212121. doi: 10.1371/journal.pone.0212121 (PMC6481805; doi:10.1371/journal.pone.0212121)
Supplement: S1 Table — (PDF) [file pone.0212121.s004.pdf]

S1 Table. Nonsense variants in human *BMP4* and *Danio rerio bmp4*

**Human gene/ Transcript**	**Inheritance Pattern**	**Nucleotide/protein alteration**	**Stop Codon**	**Predicted effect**	**Reference**
BMP4/NM_001202.3	AD[a]	c.592C>T/ p.Arg198*	TGA	LOF[b]	[19]
BMP4/NM_001202.3	AR[c]	c.637G>T/ p.Glu213*	TAA	LOF	-
**Zebrafish gene/ Transcript**	**Inheritance Pattern**	**Nucleotide/protein alteration**	**Stop Codon**	**Predicted effect**	**Reference**
bmp4/NM_131342	AR	c.625G>T/ p.Glu209*	TAA	LOF	[21]

AD[a] = autosomal dominant; LOF[b] = loss of function; AR[c] = autosomal recessive.
