# Supplementary material for: Use of PTC124 for nonsense suppression therapy targeting BMP4 nonsense variants in vitro and the bmp4st72 allele in zebrafish
Source: PLoS One. 2019 Apr 24;14(4):e0212121. doi: 10.1371/journal.pone.0212121 (PMC6481805; doi:10.1371/journal.pone.0212121)
Supplement: S2 Table — The average 450 nm optical density (OD) for the empty vector wells was determined for each PTC124 dose and subtracted from each well with wildtype or mutant constructs. Expression in the empty vector wells was normalized to 0 and the results from each data point were plotted without scaling. (PDF) [file pone.0212121.s005.pdf]

S2 Table. Data from In-cell ELISA assay after treatment of 293T/17 cells with 0-20 μM PTC124

	Mean[a] 0 μM	Mean 10 μM	Mean 20 μM	SEM[b] 0 μM	SEM 10 μM	SEM 20 μM	p value[c]
BMP4 wildtype	0.799	0.894	0.988	0.072	0.092	0.103	p = 0.21
BMP4 p.Arg198*	0.017	0.045	0.032	0.030	0.034	0.050	p = 0.09
BMP4 p.Glu213*	0.448	0.438	0.616	0.042	0.047	0.062	p = 0.81

The average 450 nm optical density (OD) for the empty vector wells was determined for each PTC124 dose and subtracted from each well with wildtype or mutant constructs. Expression in the empty vector wells was normalized to 0 and the results from each data point were plotted without scaling.

Mean[a] = each mean is the mean of three independent experiments using 5 replicates per data point. SEM[b] = standard error of the mean for three independent experiments using 5 replicates per data point. p value[c] = significance of comparison of means for 0 and 20 μM for each construct.
