# Supplementary material for: Use of PTC124 for nonsense suppression therapy targeting BMP4 nonsense variants in vitro and the bmp4st72 allele in zebrafish
Source: PLoS One. 2019 Apr 24;14(4):e0212121. doi: 10.1371/journal.pone.0212121 (PMC6481805; doi:10.1371/journal.pone.0212121)
Supplement: S3 Table — Larvae were not dechorionated and PTC124 treatment was started at 0 hpf. Each measurement is the mean of 3 or 4 independent experiments; data includes results from experiments not included in Table 1 due to low penetrance of ventroposterior defects in control homozygous larvae in the experiment. (PDF) [file pone.0212121.s006.pdf]

S3 Table. PTC124 treatment at 1 µM or 2 µM increases non-specific toxicity in treated larvae compared to untreated larvae at 72 hours post fertilization (hpf) in *bmp4*$^{st72/+}$ in-crossed zebrafish.

PTC124 concentration	Larvae with toxicity/ total larvae	%
0 µM	12/214	5.6%
1 µM	25/218	11.5%
2 µM	39/137	28.5%
