# Supplementary material for: Use of PTC124 for nonsense suppression therapy targeting BMP4 nonsense variants in vitro and the bmp4st72 allele in zebrafish
Source: PLoS One. 2019 Apr 24;14(4):e0212121. doi: 10.1371/journal.pone.0212121 (PMC6481805; doi:10.1371/journal.pone.0212121)
Supplement: S4 Table — Larvae were dechorionated at 6–8 hpf and PTC124 treatment was started at 6–8 hpf. Each measurement is the mean of 3 independent experiments. (PDF) [file pone.0212121.s007.pdf]

S4 Table. PTC124 treatment at 0.25 μM or 0.5 μM does not increase non-specific toxicity in dechorionated larvae compared to untreated larvae at 72 hours post fertilization (hpf) in *bmp4*$^{st72/+}$ in-crossed zebrafish

PTC124 concentration	Larvae with toxicity/ total larvae	%
0 μM	17/149	11.41%
0.25 μM	5/128	3.91%
0.5 μM	6/136	4.41%
